# Pulmonary involvement in Kaposi sarcoma: correlation between imaging and pathology

**DOI:** 10.1186/1750-1172-4-18

**Published:** 2009-07-14

**Authors:** Taisa Davaus Gasparetto, Edson Marchiori, Sílvia Lourenço, Gláucia Zanetti, Alberto Domingues Vianna, Alair ASMD Santos, Luiz Felipe Nobre

**Affiliations:** 1 Department of Radiology, Fluminense Federal University, Rio de Janeiro, Brazil; 2 Department of Radiology, Faculty of Medicine, Federal University of Rio de Janeiro, Rio de Janeiro, Brazil; 3 Faculty of Medicine, Santa Catarina Federal University, Florianópolis, Brazil

## Abstract

Kaposi sarcoma is a low-grade mesenchymal tumor involving blood and lymphatic vessels. There are four variants of this disease, each presenting a different clinical manifestation: classic or sporadic, African or endemic, organ transplant-related or iatrogenic, and AIDS-related or epidemic. Kaposi sarcoma is the most common tumor among patients with HIV infection, occurring predominantly in homosexual or bisexual men. The pulmonary involvement in Kaposi sarcoma occurs commonly in critically immunosupressed patients who commonly have had preceding mucocutaneous or digestive involvement.

The etiology of Kaposi sarcoma is not precisely established; genetic, hormonal, and immune factors, as well as infectious agents, have all been implicated. There is evidence from epidemiologic, serologic, and molecular studies that Kaposi sarcoma is associated with human herpes virus type 8 infection. The disease starts as a reactive polyclonal angioproliferative response towards this virus, in which polyclonal cells change to form oligoclonal cell populations that expand and undergo malignant transformation.

The diagnosis of pulmonary involvement in Kaposi sarcoma usually can be made by a combination of clinical, radiographic, and laboratory findings, together with the results of bronchoscopy and transbronchial biopsy. Chest high-resolution computed tomography scans commonly reveal peribronchovascular and interlobular septal thickening, bilateral and symmetric ill-defined nodules in a peribronchovascular distribution, fissural nodularity, mediastinal adenopathies, and pleural effusions. Correlation between the high-resolution computed tomography findings and the pathology revealed by histopathological analysis demonstrate that the areas of central peribronchovascular infiltration represent tumor growth involving the bronchovascular bundles, with nodules corresponding to proliferations of neoplastic cells into the pulmonary parenchyma. The interlobular septal thickening may represent edema or tumor infiltration, and areas of ground-glass attenuation correspond to edema and the filling of air spaces with blood. These findings are a result of the propensity of Kaposi sarcoma to grow in the peribronchial and perivascular axial interstitial spaces, often as continuous sheets of tumor tissue.

In conclusion, radiological findings can play a major role in the diagnosis of pulmonary Kaposi sarcoma since characteristic patterns may be observed. The presence of these patterns in patients with AIDS is highly suggestive of Kaposi sarcoma.

## Review

### Introduction

Kaposi sarcoma (KS) was first described by Moritz von Kaposi in 1872 as a low-grade mesenchymal tumor involving blood and lymphatic vessels. The mucocutaneous sites are primarily affected, typically the skin of the lower extremities, face, trunk, genitalia, and oropharyngeal mucosa; other organs are involved in the disseminated form of the disease [1,3]. This disease is recognized to arise as four variants, each presenting a different clinical manifestation: classic or sporadic, African or endemic, organ transplant-related or iatrogenic, and acquired immunodeficiency syndrome (AIDS)-related or epidemic [1,4,5].

KS is the most common tumor among patients with human immunodeficiency virus (HIV) infection, occurring predominantly in homosexual or bisexual men [6,7]. Also, an increasing number of reports describe KS as a complication of solid organ transplantation [1,5,8,9]. Pulmonary involvement generally occurs in severely immunosupressed patients who already have mucocutaneous or digestive involvement [6].

### Epidemiology

KS is one of the major complications of AIDS [10]. In industrialized countries, KS occurs in patients of all ages, primarily homosexual males; it is much less common in heterosexual males, being observed in less than 10% of patients in other groups at risk for HIV infection [6,11,12]. The use of highly active antiretroviral therapies (HAART) has lead to a decline in the incidence of KS [6,13,14]. Recent studies showed that the incidence of KS decreased from 30/1000 patient-years in the pre-HAART era to 0.03/1000 patient-years in the HAART era [15].

Critical immunosupression in patients with mucocutaneous KS commonly leads to pulmonary involvement. Thoracic disease is found in about 45% of patients with cutaneous AIDS-related KS, and in about 15% of patients without mucocutaneous involvement [5]. It must be noted that these high rates of pulmonary disease refer to autopsy findings, in the pre-HAART era. Currently, after the introduction of this therapy, pulmonary involvement has probably become much less frequent. Palmieri et al [10]. studied the clinicopathological differences between patients with and without pulmonary KS diagnosed in the era of HAART. The authors concluded that in HIV-1-infected patients diagnosed with KS, pulmonary involvement was associated with a low CD4 cell count, suggesting that pulmonary KS may be related to late presentation of HIV disease [10].

### Pathology and Pathogenesis

The etiology of KS is not precisely established; genetic, hormonal, and immune factors, as well as infectious agents, have all been implicated. There is evidence from epidemiologic, serologic, and molecular studies that KS is associated with human herpes virus type 8 (HHV8) infection [1,6,13]. In addition, other agents such as cytokine-induced growth factors have been linked to the development of the disease [1,5].

The presence of KS associated with HHV-8 and host immunosuppression are considered the major factors that promote tumor development [8,16-18]. The disease starts as a reactive polyclonal angioproliferative response towards HHV-8, in which polyclonal cells change to form oligoclonal cell populations that expand and undergo malignant transformation [19].

The histopathologic process of the disease is believed to start in the sub epithelial connective tissue, extending in the direction of the epithelium. A developed lesion consists of interwoven bands of spindle cells and vascular structures grouped in a network of reticular and collagen fibers. Erythrocytes are seen within these vascular structures and interspersed between spindle cells. The vascular component appears as small capillaries or slit-like spaces between the spindle cells [11].

### Clinical Manifestations

Generally, patients with lung KS have previously treated cutaneous lesions or other visceral involvement, for example in the gastrointestinal tract. Nonetheless, cutaneous involvement is absent in 5–23% of patients with symptomatic pulmonary KS [11]. The most common symptoms are progressive dyspnea, non-productive cough, and fever. Although some authors have described fever as a common finding, it cannot be distinguished from superimposed infection [11,20,21]. Pleural effusion with chest pain, hypoxemia, and acute respiratory failure requiring mechanical ventilation have also been reported. Physical examination of the thorax is usually normal, but non-specific signs such as crackles, wheezing, and stridor may be present [11].

Four clinical variants of KS have been described [1,5]. The histological findings are identical between the variants, but each affects distinct populations and shows characteristic sites of involvement and rates of progression. The classic variant primarily affects elderly men of Eastern European and Mediterranean origin, and Ashkenazi Jews; it is more common in men than in women, by a ratio as high as 15 to 1 [5,8]. There is a cutaneous asymptomatic form of the disease, presenting with multiple firm, purple-blue or reddish-brown plaques and nodules, distributed mainly in the lower limbs. Untreated lesions evolve to plaques and ulcerated nodules, with venous stasis and lymphedema. A second type of the classic variant of KS comprises a more aggressive form, with rapid progression, presenting with disseminated mucocutaneous and visceral lesions [5,8]. The second variant, African or endemic KS, affects men in East and Central Africa in the 4th decade of life, with a male-female ratio of 17:1. This form of KS is clinically similar to the classic form, although with a more aggressive variant that responds poorly to conventional treatment [5,8]. The third variant, KS related to solid organ-transplantation, is considered a complication related to chronic drug-induced immunosuppression. The median interval from organ transplantation to diagnosis is 29 to 31 months. This type of KS tends to be aggressive, involving lymph nodes, mucosa, and visceral organs in about half of the patients, sometimes in the absence of skin lesions [8]. AIDS-related KS, the fourth variant, is an aggressive epidemic form involving lymph nodes, viscera, and mucosa as well as skin. This type of KS affects predominantly homosexual men with AIDS and may be fatal in the absence of HAART and KS-specific treatment. HAART has been shown to have a dramatic effect on KS in patients with AIDS. Since the introduction of this treatment, a substantial decrease in the incidence and prevalence of HIV-KS as well as regression of established KS lesions have been reported [22,23]. The use of HAART increases overall survival in patients with KS and is associated with an 80% reduction in the risk of death among KS patients [24]. About 20% of deaths are related to complications of the disease itself (upper airway obstruction, hemorrhage, or parenchymal destruction), but the majority of deaths are related to other factors (eg, concomitant infection) [5].

### Bronchoscopy

The extent of tracheobronchial KS ranges from isolated tracheal lesions to diffuse and/or extensive tracheobronchial involvement. Endobronchial lesions may narrow and partially obstruct the airways. The bronchoscopic appearance of endobronchial KS is considered to be characteristic enough to allow a diagnosis. When bronchoscopic examination identifies KS lesions below the level of the carina, accompanying parenchymal abnormalities are seen, as a rule, on chest radiography or CT. Thus, the appearance of characteristic tracheobronchial KS lesions is sufficient to make a presumptive diagnosis of pulmonary KS [25,26].

### Treatment

There is increasing evidence that HAART and an improved immune response are associated with complete or partial regression of KS lesions, a decrease in the number of patients suffering from KS, improved survival, and protection of HIV-infected patients against the development of KS [13,14]. In patients using HAART, regression of the lesions correlates with a decrease in plasma HIV load and improved immune response [13]. Some studies showed that HAART alone can lead to stabilization and regression of KS, often eliminating the need of chemotherapy and radiation therapy, and prolonging remission among patients with a complete response. Patients with pulmonary KS using HAART showed a median survival time of 1.6 years compared with a median survival time of 4 months in the pre-HAART era [10].

### Imaging Diagnosis: Chest X-Ray and CT Findings

The diagnosis of pulmonary involvement in KS usually can be made by a combination of clinical, radiographic, and laboratory findings, together with the results of bronchoscopy and transbronchial biopsy [27]. KS may involve the tracheo-bronchial tree, the pulmonary parenchyma, and the pleura [20,21,28]. Pleural involvement generally occurs only in the presence of parenchymal abnormalities. Also, the presence of lesions in the bronchial tree below the carina is commonly accompanied by parenchymal findings [28,29]. Thus, there is consensus that the presence of lesions characteristic of KS in the tracheo-bronchial tree is sufficient for a presumptive diagnosis of pulmonary KS.

**Figure 1:**
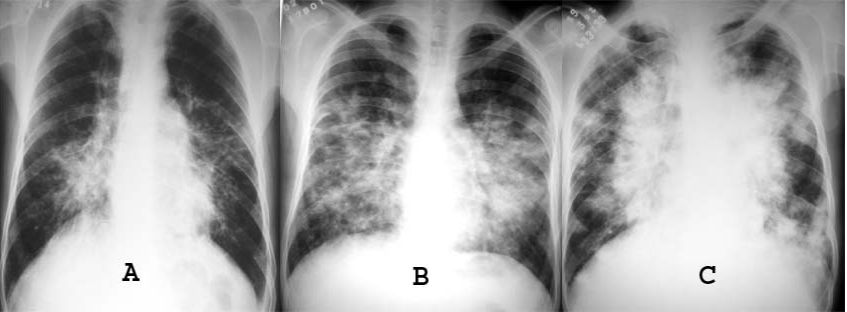
**A to C. Chest X-rays of three patients with pulmonary KS showing bilateral paracardiac infiltration**. Confluent lesions are most evident in C.

**Figure 2:**
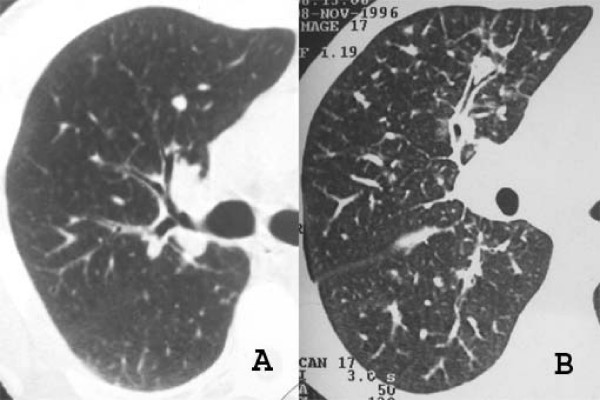
**A and B. High-resolution CT scans of two patients with pulmonary KS showing peribronchovascular thickening and irregular narrowing of the bronchial lumen**.

**Figure 3:**
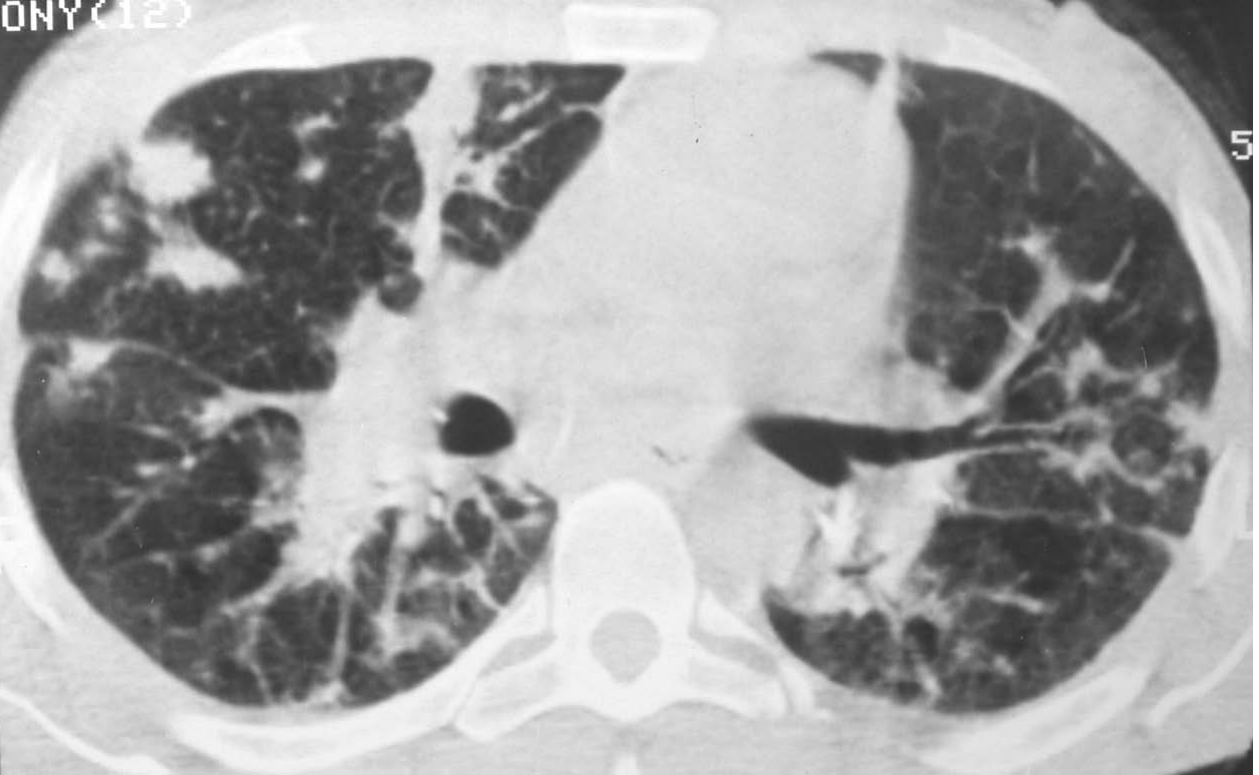
**High-resolution CT scan at the level of the main bronchi of a patient with pulmonary KS, showing diffuse ill-defined large nodules and paracardiac peribronchovascular thickening**.

**Figure 4:**
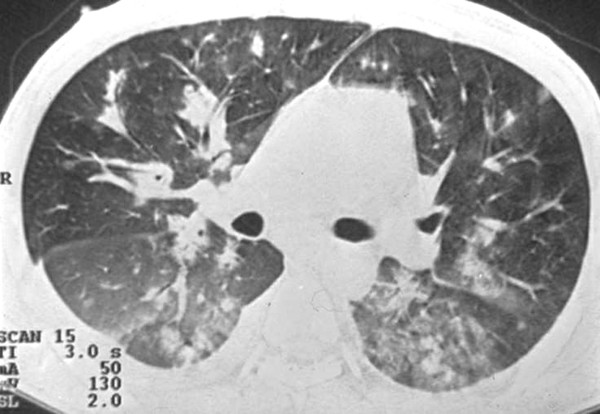
**High-resolution CT scan of a patient with pulmonary KS at the level of the main bronchi shows ground-glass attenuation areas in the posterior regions of both lungs, which correspond to pulmonary hemorrhage**. Peribronchovascular thickening is observed in the right lung, as well as bilateral pleural effusion.

**Figure 5:**
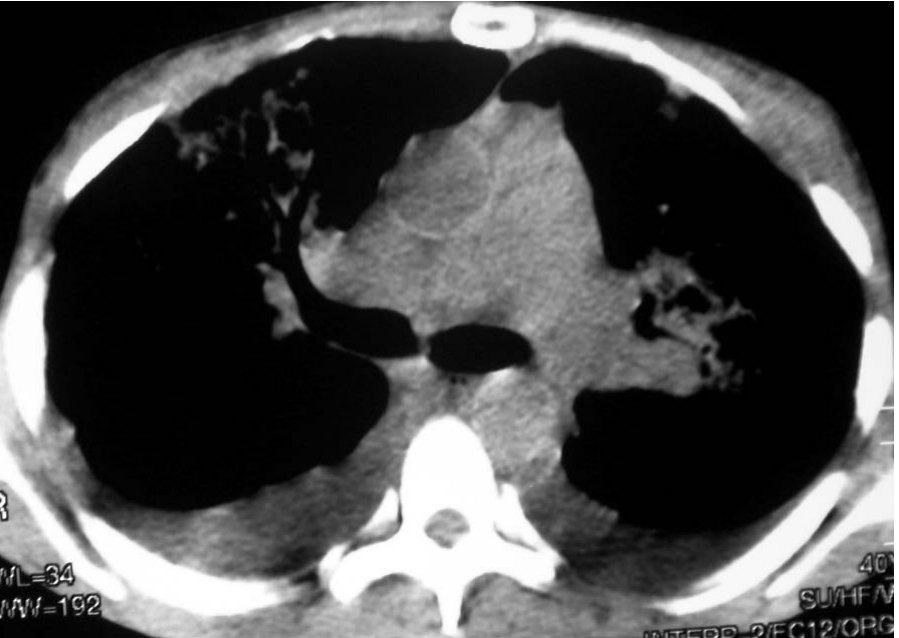
**CT scan (mediastinal window) at the level of the carina of a patient with pulmonary KS shows bilateral pleural effusion**.

Chest radiography may demonstrate middle to lower lung zone reticular opacities and parenchymal nodules with a bronchovascular distribution that may progress to consolidation, peribronchial cuffing, Kerley B lines, pleural collections, and hilar or mediastinal adenopathies [5] (Figure 1). HRCT scans have more specificity and sensitivity than the chest X-rays, and can provide important data leading to suspicion of the diagnosis of pulmonary KS. The most frequent CT finding is interstitial thickening, involving the peribronchovascular sheaths, often beginning in the peri-hilar regions and then progressing to the periphery. The peribronchovascular thickening may be associated with irregular narrowing of the bronchial lumen by mucous lesions (Figure 2). The involvement is predominant in the middle and lower thirds of the lungs, preserving the upper lobes of the lungs. The confluence of the lesions leads to progressive air-space consolidation, possibly with a component of airway obstruction. Other HRCT scan findings are interlobular septal thickening, usually preserving the lobular architecture and simulating carcinomatous lymphangitis; large parenchymal nodules with irregular, poorly defined, and spiculated borders (Figure 3), some of them with a perinodular ground glass halo sign and air bronchograms; fissural nodularity; ground-glass opacities (Figure 4); mediastinal adenopathies; and pleural effusions [5,11,20,21,27,28,30-32] (Figure 5).

The sensitivity of HRCT in the diagnosis of endobronchial KS is not high, but tumors large enough to cause stridor in the upper airways or atelectasis in small segmental or lobar bronchi are generally seen as filling defects in the endobronchial lumen. Endoluminal lesions are infrequently detected by HRCT; thickening of the bronchial walls and interstitium, representing tumor infiltration along the interstitium, is much more common [20,33].

### Correlation of HRCT and Pathologic Findings

**Figure 6:**
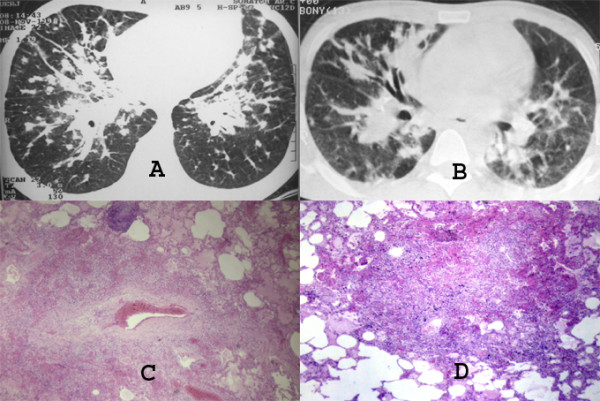
**A to D. High-resolution CT scans (A and B) of two patients with pulmonary KS that demonstrate marked peribronchovascular and interlobular septal thickening and the presence of small parenchimal nodules**. Photomicrographs of histologic specimen show tumor cells infiltrating the periarteriolar connective tissue (C), and a neoplastic parenchymal nodule with indistinct borders (D) (HE, ×40).

**Figure 7:**
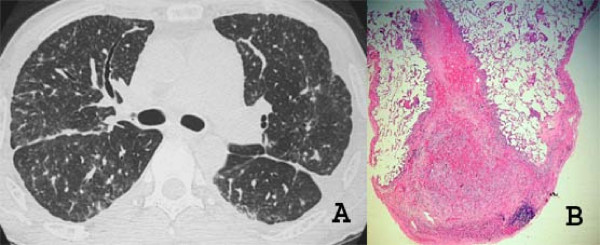
**A and B. High-resolution CT scan at the level of the main bronchi (A) of a patient with pulmonary KS demonstrates irregularity of the pleural surfaces and nodularity of the oblique fissures bilaterally**. Photomicrograph of histologic section (B) demonstrates a neoplastic nodule adjacent to the pleural surface (HE, ×40).

**Figure 8:**
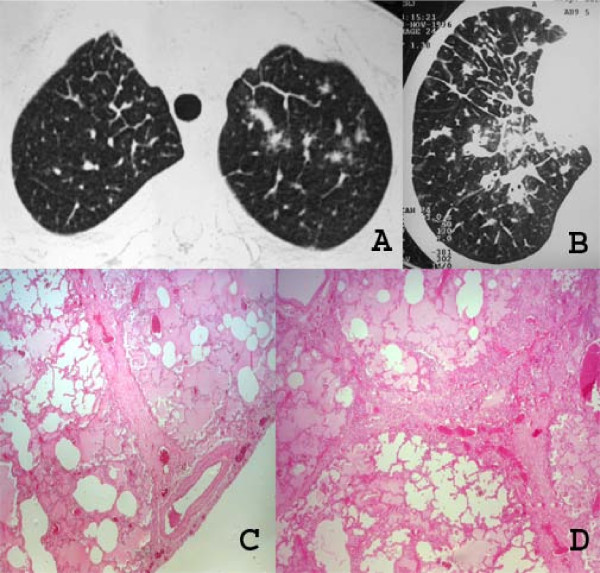
**A to D. High-resolution CT scans at the level of the upper lobe (A) and the lower lobe (B), of two patients with pulmonary KS, show extensive interlobular septal and peribronchovascular thickening**. Photomicrographs of histologic specimens (C and D) show thickening of interlobular septa due to edema and tumor cells infiltration (HE, ×40).

The imaging findings of pulmonary KS reflect the propensity of KS to grow in the peribronchial and perivascular axial interstitial spaces extending to the peripheral regions, often as continuous sheets of tumor tissue (Figure 6). Thus central peribronchovascular infiltration represents confluent tumor, and irregular nodular opacities are interpreted as tumor proliferation extending into the parenchyma. Cellular infiltration of the pulmonary parenchyma begins with invasion of the interstitial space, with occupation of the peribronchovascular tissue along the pulmonary vessels and the pleural surface. The tumor cells progress from these locations to the adjacent alveolar spaces, filling the alveoli and forming solid irregular nodules. The peripheral interstitial compartment and visceral pleural surfaces can also become affected (Figure 7). The interlobular septa may be thickened by edema or diffuse infiltration by tumor cells [28,31,34,35] (Figure 8).

**Figure 9:**
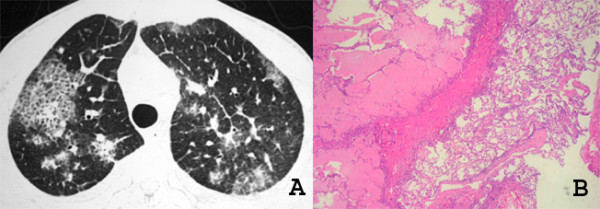
**A and B. High-resolution CT scan (A) shows areas of ground-glass attenuation and interlobular septal thickening in the upper lobes**. A crazy-paving pattern is observed in the right upper lobe. Photomicrograph of histologic section (B) demonstrates infiltration of the interlobular septa due to edema and neoplastic cells and also edema filling the alveolar airspace (HE, ×40).

Silva Filho et al. [32] reported the association of the "crazy-paving" pattern with peribronchovascular thickening in patients with pulmonary KS. This pathological correlation demonstrated that the areas of ground-glass attenuation represented edema and filling of air spaces with blood, and that the interlobular septal thickening was related to the infiltration of the interlobular septa by neoplastic cells (Figure 9).

## Conclusion

In conclusion, tomographic findings can play an important role in the diagnosis of pulmonary KS, since characteristic patterns may be observed. The most frequent chest CT findings are thickening of the peribronchovascular interstitium, particularly in the axial portions; irregular and poorly defined nodules; and interlobular septal thickening. The presence of these findings in patients with AIDS is highly suggestive of KS. Correct interpretation of the HRCT patterns, understanding of the histopathological appearance, and association of the histopathological and radiological findings can be very useful to the radiologist in the correct diagnosis of pulmonary KS.

## Abbreviations

KS: Kaposi's sarcoma; AIDS: acquired immunodeficiency syndrome; HIV: human immunodeficiency virus; HHV8: human herpes virus type 8; HAART: highly active antiretroviral therapies.

## Competing interests

The authors declare that they have no competing interests.

## Authors' contributions

TDG conceived the study. SLN, ADV and AASDS performed the literature review. TDG, GZ and EM edit and coordinated the manuscript. All authors read and approved the final manuscript.

## References

[B1] Martinez S, McAdams HP, Youens KE (2008). Kaposi sarcoma after bilateral lung transplantation. J Thorac Imaging.

[B2] Pantanowitz L, Dezube BJ (2008). Kaposi sarcoma in unusual locations. BMC Cancer.

[B3] Pantanowitz L, Mullen J, Dezube BJ (2008). Primary Kaposi sarcoma of the subcutaneous tissue. World J Surg Oncol.

[B4] Grayson W, Pantanowitz L (2008). Histological variants of cutaneous Kaposi sarcoma. Diagn Pathol.

[B5] Restrepo CS, Martinez S, Lemos JA, Carrillo JA, Lemos DF, Ojeda P (2006). Imaging manifestations of Kaposi sarcoma. Radiographics.

[B6] Godoy MC, Rouse H, Brown JA, Phillips P, Forrest DM, Muller NL (2007). Imaging features of pulmonary Kaposi sarcoma-associated immune reconstitution syndrome. AJR Am J Roentgenol.

[B7] Mbulaiteye SM, Parkin DM, Rabkin CS (2003). Epidemiology of AIDS-related malignancies an international perspective. Hematol Oncol Clin North Am.

[B8] Antman K, Chang Y (2000). Kaposi's sarcoma. N Engl J Med.

[B9] Volkow P, Zinser JW, Correa-Rotter R (2007). Molecularly targeted therapy for Kaposi's sarcoma in a kidney transplant patient: case report, "what worked and what did not". BMC Nephrol.

[B10] Palmieri C, Dhillon T, Thirlwell C, Newsom-Davis T, Young AM, Nelson M (2006). Pulmonary Kaposi sarcoma in the era of highly active antiretroviral therapy. HIV Med.

[B11] Cadranel J, Naccache J, Wislez M, Mayaud C (1999). Pulmonary malignancies in the immunocompromised patient. Respiration.

[B12] Tappero JW, Conant MA, Wolfe SF, Berger TG (1993). Kaposi's sarcoma. Epidemiology, pathogenesis, histology, clinical spectrum, staging criteria and therapy. J Am Acad Dermatol.

[B13] Kanmogne GD (2005). Noninfectious pulmonary complications of HIV/AIDS. Curr Opin Pulm Med.

[B14] Feller L, Lemmer J (2008). Insights into pathogenic events of HIV-associated Kaposi sarcoma and immune reconstitution syndrome related Kaposi sarcoma. Infect Agent Cancer.

[B15] Portsmouth S, Stebbing J, Gill J, Mandalia S, Bower M, Nelson M (2003). A comparison of regimens based on non-nucleoside reverse transcriptase inhibitors or protease inhibitors in preventing Kaposi's sarcoma. AIDS.

[B16] Colman R, Blackbourn DJ (2008). Risk factors in the development of Kaposi's sarcoma. AIDS.

[B17] Mwakigonja AR, Pyakurel P, Kokhaei P, Pak F, Lema LK, Kaaya EE (2008). Human herpesvirus-8 (HHV-8) sero-detection and HIV association in Kaposi's sarcoma (KS), non-KS tumors and non-neoplastic conditions. Infect Agent Cancer.

[B18] Pyakurel P, Pak F, Mwakigonja AR, Kaaya E, Biberfeld P (2007). KSHV/HHV-8 and HIV infection in Kaposi's sarcoma development. Infect Agent Cancer.

[B19] Wood NH, Feller L (2008). The malignant potential of HIV-associated Kaposi sarcoma. Cancer Cell Int.

[B20] McGuinness G (1997). Changing trends in the pulmonary manifestations of AIDS. Radiol Clin North Am.

[B21] Huang L, Schnapp LM, Gruden JF, Hopewell PC, Stansell JD (1996). Presentation of AIDS-related pulmonary Kaposi's sarcoma diagnosed by bronchoscopy. Am J Respir Crit Care Med.

[B22] Haramati LB, Jenny-Avital ER, Alterman DD (2007). Thoracic manifestations of immune restoration syndromes in AIDS. J Thorac Imaging.

[B23] Feller L, Anagnostopoulos C, Wood NH, Bouckaert M, Raubenheimer EJ, Lemmer J (2008). Human immunodeficiency virus-associated Kaposi sarcoma as an immune reconstitution inflammatory syndrome: a literature review and case report. J Periodontol.

[B24] Nasti G, Talamini R, Antinori A, Martellotta F, Jacchetti G, Chiodo F (2003). AIDS-related Kaposi's Sarcoma: evaluation of potential new prognostic factors and assessment of the AIDS Clinical Trial Group Staging System in the Haart Era – the Italian Cooperative Group on AIDS and Tumors and the Italian Cohort of Patients Naive From Antiretrovirals. J Clin Oncol.

[B25] Yoo DJ, Lee KH, Munderi P, Shin KC, Lee JK (2005). Clinical and bronchoscopic findings in Ugandans with pulmonary Kaposi's sarcoma. Korean J Intern Med.

[B26] Miller RF, Tomlinson MC, Cottrill CP, Donald JJ, Spittle MF, Semple SJ (1992). Bronchopulmonary Kaposi's sarcoma in patients with AIDS. Thorax.

[B27] Kang EY, Staples CA, McGuinness G, Primack SL, Muller NL (1996). Detection and differential diagnosis of pulmonary infections and tumors in patients with AIDS: value of chest radiography versus CT. AJR Am J Roentgenol.

[B28] Gruden JF, Huang L, Webb WR, Gamsu G, Hopewell PC, Sides DM (1995). AIDS-related Kaposi sarcoma of the lung: radiographic findings and staging system with bronchoscopic correlation. Radiology.

[B29] Meduri GU, Stover DE, Lee M, Myskowski PL, Caravelli JF, Zaman MB (1986). Pulmonary Kaposi's sarcoma in the acquired immune deficiency syndrome. Clinical, radiographic, and pathologic manifestations. Am J Med.

[B30] Khalil AM, Carette MF, Cadranel JL, Mayaud CM, Bigot JM (1995). Intrathoracic Kaposi's sarcoma. CT findings. Chest.

[B31] Marchiori E, Muller NL, Soares Souza A, Escuissato DL, Gasparetto EL, Franquet T (2005). Pulmonary disease in patients with AIDS: high-resolution CT and pathologic findings. AJR Am J Roentgenol.

[B32] da Silva Filho FP, Marchiori E, Valiante PM, Escuissato DL, Gasparetto TD (2008). AIDS-related Kaposi sarcoma of the lung presenting with a "crazy-paving" pattern on high-resolution CT: imaging and pathologic findings. J Thorac Imaging.

[B33] McGuinness G, Gruden JF, Bhalla M, Harkin TJ, Jagirdar JS, Naidich DP (1997). AIDS-related airway disease. AJR Am J Roentgenol.

[B34] Davis SD, Henschke CI, Chamides BK, Westcott JL (1987). Intrathoracic Kaposi sarcoma in AIDS patients: radiographic-pathologic correlation. Radiology.

[B35] Scully RE, Mark EJ, McNeely WF, Ebeling SH, Phillips LD (1997). Case records of the Massachusetts General Hospital. Weekly clinicopathological exercises. Case 20,-1997. A 74-year-old man with progressive cough, dyspnea, and pleural thickening. N Engl J Med.

